# Proteomic diversity of Russell's viper venom: exploring PLA2 isoforms, pharmacological effects, and inhibitory approaches

**DOI:** 10.1007/s00204-024-03849-5

**Published:** 2024-08-24

**Authors:** Kishore Srinivasan, Madhavan Nampoothiri, Shweta Khandibharad, Shailza Singh, Akshatha Ganesh Nayak, Raghu Chandrashekar Hariharapura

**Affiliations:** 1 Department of Pharmaceutical Biotechnology, Manipal College of Pharmaceutical Sciences, Manipal Academy of Higher Education, Manipal, Karnataka India; 2 Department of Pharmacology, Manipal College of Pharmaceutical Sciences, Manipal Academy of Higher Education, Manipal, Karnataka India; 3 National Centre for Cell Science, NCCS Complex, Savitribai Phule Pune University Campus, Ganeshkhind, Pune, Maharashtra India; 4 Division of Biochemistry, Department of Basic Medical Sciences, Manipal Academy of Higher Education, Manipal, Karnataka India

**Keywords:** Russell’s viper, *Daboia russelii*, Phospholipase A2, Antivenoms, Low-molecular-weight toxins

## Abstract

Snakebite envenomation is a serious health concern in tropical regions, resulting in high mortality. The World Health Organization (WHO) has declared it a neglected tropical disease and is working on strategies to reduce mortality. Russell’s viper (*Daboia russelii*) is one of the most abundant venomous snakes found across Southeast Asia. Proteomic analysis of Russell’s viper venom has demonstrated variation, with phospholipase A2 (PLA2) being the most abundant toxin across geographic boundaries. PLA2, a major constituent of the low-molecular-weight fraction of snake venom, hydrolyses phospholipids at the sn-2 position, releasing arachidonic acid and lysophospholipids. They are reported to cause various pharmacological effects, including hemolysis, anticoagulation, neurotoxicity, myotoxicity, and oedema. Though administration of antivenoms (ASV) is the primary treatment for envenomation, it has many drawbacks. Besides causing hypersensitivity reactions and life-threatening anaphylaxis, treatment with ASV is further complicated due to its inability to neutralize low-molecular-weight toxins. Thus, there is a greater need to produce next-generation antivenoms that can target specific toxins in the venom. In this review, we explored the classification of Russell’s viper and the variation in its proteomic profile across Southeast Asia to date. In addition, we have also summarized the mechanism of action of PLA2 and discussed various isoforms of PLA2 found across different regions with their respective pharmacological effects. Finally, the drawbacks of commercially available antivenoms and the molecules investigated for inhibiting the low-molecular-weight toxin, PLA2 are discussed.

**Supplementary Information:**

The online version contains supplementary material available at 10.1007/s00204-024-03849-5.

## Introduction

Snakebite envenomation is a major cause of mortality in tropical regions, with global statistics reporting nearly 1.8–2.7 million envenomation cases annually, resulting in approximately 81,410–1,37,880 deaths. In response to this public threat, the World Health Organization (WHO) declared snakebite envenomation, a neglected tropical disease in 2017, intending to reduce morbidity and mortality by 50% before 2030 (World Health Organization 2023). Venomous snakebites can cause local tissue damage namely oedema and hemorrhage, as well as systemic effects such as hepatotoxicity, nephrotoxicity, cardiotoxicity, myotoxicity, and neurotoxicity, which can lead to paralysis. Snakebites have immediate and serious effects on children compared to adults due to their smaller body mass (World Health Organization 2023). Among the various snakes reported worldwide, Russell’s viper is one of the most lethal species and is mainly found in Southeast Asia. In India, it accounts for nearly 42% of reported snakebite cases (Suraweera et al. 2020). In general, snake venom is composed mainly of proteins and peptides (90–95%), which are further classified into enzymatic and nonenzymatic components. Phospholipase A2 (PLA2), snake venom metalloproteinases (SVMPs), snake venom serine proteases (SVSPs), and L-amino acid oxidases (LAAOs) are the major enzymatic components, while kunitz-type serine protease inhibitors (KSPIs), snaclec/C-type lectins/C-type lectin-like (CTLs) and cysteine-rich secretory proteins (CRISPs) are the major nonenzymatic components (Munawar et al. 2018). Studies have reported the venom composition to vary among snakes across different biogeographical regions. Half a decade ago, a comprehensive review focused on the proteomic venom composition of Russell’s viper across the Indian subcontinent (Kalita et al. 2018a). Thereafter, several research groups have explored the proteomic profiles of Russell’s viper venom indigenous to other geographical locations across Southeast Asia, including Indonesia (Lingam et al. 2020), Bangladesh (Pla et al. 2019; Yasmin et al. 2024), and Thailand (Lingam et al. 2020). To date, antivenom administration remains the standard treatment for snake bite envenomation globally. However, the antivenoms are ineffective against some snake species and even lack efficacy against specific snakes against which the antivenom was produced. This emphasizes the need for developing next generation antivenoms wherein individual toxins are targeted (Vanuopadath et al. 2023). This review aims to present the current proteomic profiles of Russell's viper venoms in Southeast Asia. It will also explore the mechanism of action of the prevalent toxin PLA2, its isoforms, and their respective pharmacological functions, providing a comprehensive understanding of the diversity of the venom. In contrast to typical reviews that provide a broad overview, this review will specifically focus on the drawbacks of commercial antivenoms and the current strategies employed for developing next-generation antivenoms tailored against Russell’s viper venom.

## Russell’s viper and classification

Russell’s viper (*Daboia russelii*), which belongs to the Viperidae family, was named after Dr. Patrick Russell. The genus name “Daboia” originated from a Hindi word meaning lurker, as it lies hidden. They have a flattened and triangular head, blunt and rounded snout, and keeled scales. Their body colour is yellowish to brown, and they possess patterns made of dark, round to oblong blotches with black and white edges (Whitaker 2006). They are widespread across various regions of Southeast Asia, spanning India, Pakistan, Sri Lanka, China, Bangladesh, Myanmar, Taiwan, and Indonesia. The species has been systematically grouped into five subspecies, each differentiated by variation in body colouration and markings: (1) *Daboia russelii* (found in India, Pakistan, Nepal, and Bangladesh); (2) *Daboia russelii pulchella* (native to Sri Lanka); (3) *Daboia russelii siamensis* (distributed in Thailand, Myanmar, and China); (4) *Daboia russelii formosensis* (located in Taiwan); and (5) *Daboia russelii limitis* (identified in Indonesia) (Warrell 1989). However, through a detailed investigation of morphological features and mitochondrial DNA, Thorpe and group categorized Russell’s vipers into two distinct species: *Daboia russelii* (found in the west of the Bay of Bengal) and *Daboia siamensis* (found in the east of the Bay of Bengal) (Thorpe et al. 2007). *Daboia russelii* encompasses species found across India, Sri Lanka, Pakistan, and Bangladesh, while *Daboia siamensis* includes species inhabiting southern China, Taiwan, and Indonesia.

## Proteomic profile of Russell’s viper

Exploring the venom composition is crucial for understanding the differences among Russell’s viper species. The proteomics-based approach is by far the most widely used method to study venom composition, although tissue-based genomics and transcriptomics have gained prominence in recent years. The standard proteomic workflow comprises the decomplexation of venom followed by mass spectrometry analysis. The decomplexation process that facilitates the fractionation of venom is achieved through protein separation techniques such as chromatography [gel filtration, ion exchange, reversed-phase-high-performance liquid chromatography (RP-HPLC)], gel electrophoresis (1D or 2D SDS‒PAGE), or a combination of both. Subsequently, the fractionated venom is subjected to mass spectrometry analysis to determine the peptide sequence either by utilizing the traditional bottom-up approach or the evolving top-down approach. A detailed review of the various techniques adopted in proteomic studies of snake venom has been reported elsewhere (Chanda and Mukherjee 2020; Tan 2022).

Different decomplexation strategies and analyzing techniques have been used to explore the proteomic profiles of Russell’s viper. 1D and 2D SDS‒PAGE together with LC–MS/MS analysis was utilized to investigate the proteome profiles of venom from Sri Lanka (Tan et al. 2015) and Myanmar (Risch et al. 2009), respectively. On the other hand, venom from Pakistan (Mukherjee et al. 2016) and India (Kalita et al. 2017, 2018b; Sharma et al. 2015) was fractionated by gel filtration chromatography alone or in tandem with ion-exchange chromatography, which was followed by mass spectrometry analysis. At present, RP-HPLC-based decomplexation methods have been frequently used to study the proteomics profiles of venom from Pakistan (Faisal et al. 2018), India (Faisal et al. 2021), Indonesia (Lingam et al. 2020), and Sri Lanka (Faisal et al. 2021). In all these studies, the label-free spectral counting method was employed to quantify the relative abundance of toxins in the chromatographic fractions. One of the major drawbacks of using this method for venom proteome quantification is the absence of a comprehensive homolog database. On the other hand, Pla et al. (2019) used a different approach to quantify the relative abundance wherein the “percentage of total peptide bond concentration in the peak” was used.

Proteomic studies conducted across different geographical regions have highlighted variations in toxin profiles. The proteome profile of Russell’s viper venom across Southeast Asia is shown in Table 1. Toxins such as PLA2, SVMP, and SVSP were observed in the venom of all Russell’s viper species across Southeast Asia. PLA2 is the most abundant toxin with South India (Pla et al. 2019) and Sri Lanka (Faisal et al. 2021) showing the majority. However, the proteome profile of China (Tan et al. 2018) and certain studies performed in the Eastern part of India (Kalita et al. 2018b; Senji Laxme et al. 2021) and Taiwan (Tan et al. 2018) demonstrated KSPI to be an abundant toxin. Apart from PLA2, SVMP was abundant in the venom of Pakistan (Mukherjee et al. 2016), Eastern India (Kalita et al. 2018b), and Thailand (Saethang et al. 2022), while SVSP was reported to be high in Thailand (Saethang et al. 2022), Indonesia (Lingam et al. 2020), and India (Maharashtra) (Senji Laxme et al. 2021). Except in the Rusell’s viper venom from Taiwan (Sanz et al. 2018; Tan et al. 2018) and in a specific study on Thailand's venom (Lingam et al. 2020), all other studies reported the presence of LAAO. Likewise, Snaclec/C-type lectins/C-type lectin-like (CTL) was noted in all venom profiles, except in a particular South Indian study (Faisal et al. 2021). Furthermore, the proteomic profiles from Indonesia (Lingam et al. 2020), Thailand (Lingam et al. 2020; Saethang et al. 2022), and Myanmar (Risch et al. 2009) reported the absence of CRISP toxin. In addition, vascular endothelial growth factor (VEGF) and nerve growth factor (NGF) were reported to be absent in studies involving venom from Indonesia (Lingam et al. 2020) and Myanmar (Risch et al. 2009), respectively. Furthermore, only selective studies conducted in the venom of Sri Lanka (Pla et al. 2019; Faisal et al. 2021) and South India (Pla et al. 2019) showed the presence of SVMP inhibitors. Besides these toxins, phosphodiesterase (PDE), disintegrins, 5ʹ-nucleosidase (5ʹNT), and aminotransferase, were reported in small quantities and were found to vary among species. The proteomic profile of Indonesia (Lingam et al. 2020) reports 16.44% of unidentified proteins. However, there is still a high possibility for toxins found in other geographical regions but not reported in this species to be potentially present. Further investigation could help obtain a complete proteomic profile of this species.

**Table 1:** Abundance of venom toxins of Russell’s viper (*Daboia russelii*) across Southeast Asia

Venom Toxins	Country	SL	SL	SL	SI	SI	SI	SI	Pak	Pak	Pak	WI	WI	CI
	Year	2015	2019	2021	2015	2018	2019	2021	2016	2018	2019	2017	2021	2021
	Reference No.	1	2	3	4	5	2	3	6	7	2	8	9	9
Phospholipase A2 (PLA2)		35	60.1	63.92	23.8	43.25	70.6	67.5	32.8	63.8	50.4	32.5	59.05	44.94
Snake Venom Metalloprotease (SVMP)		6.9	4.8	7.34	9.5	4.63	3.4	0.19	21.8	2.5	4.8	24.8	1.29	6.51
Snake Venom Serine protease (SVSP)		16	12.8	5.48	17.5	12.86	8.5	11.86	3.2	5.5	9.1	8	27.12	12.73
Phosphodiesterase (PDE)		1.3	< 0.01	0.21	3.2	1.43	0.5	–	0.6	2.5	0.5	1.4	–	–
5'-nucleotidase (5'NT)		3	0.02	0.45	4.8	1.81	0.2	–	0.6	0.1	0.92	0.4	0.01	0.56
L- Amino Acid Oxidase (LAAO)		5.2	3.9	2.84	7.9	7.52	2.7	0.04	0.6	0.8	3.3	0.3	0.04	0.72
Snaclec/C-Type Lectins/C-Type Lectin Like (CTL)^b^		22.4	4.3	5.23	7.9	14.57	3.9	–	6.4	1.3	4.4	1.8	2.06	19.59
Kunitz-type serine proteinase inhibitor-like protein (KSPI)		4.6	2.6	3.03	4.8	1.67	2.4	5.41	28.4	16	17.8	12.5	3.71	6.57
Cysteine-rich secretory protein (CRISP)		2	3.3	5.97	11	4.94	2.1	4.98	2.6	1.3	2.3	6.8	6.47	0.16
Vascular Endothelial Growth Factor (VEGF)		–	1.8	2.16	3.2	3.68	1.9	1.55	1.5	4.3	2.7	1.8	0.01	7.58
Nerve growth factor (NGF)		3.5	< 0.05	0.97	1.6	1.64	0.02	1.68	0.4	1.1	0.3	4.8	0.02	0.18
Snake Venom Metalloproteinase Inhibitor (SVMPi)		–	2.9	2.35	–	–	3.5	–	–	–	–	–	–	–
Aminopeptidase (AP)		–	–	0.05	–	–	–	–	–	–	–	–	–	–
Disintegrin/Disintegrin-like (Dis)^c^		–	–	–	3.2	–	–	0.32	0.4	–	–	4.9	0.02	–
Aminotransferase (AMT)		–	–	–	–	–	–	–	0.2	–	–	–	–	–
Hyaluronidase (HA)		–	–	–	–	–	< 0.01	–	0.2	–	–	–	–	–
Phospholipase B (PLB)		0.1	–	–	–	0.97	–	–	–	–	–	0.1	–	–
Glutaminyl cyclase (GC)		–	0.04	–	–	0.99	< 0.05	–	–	–	0.01	–	–	–
Neurotoxic Three-FingerTtoxin (N-3FTx)		–	–	–	–	–	–	–	–	–	–	–	0.21	0.47
Cytotoxic Three-Finger Toxin (C-3FTx)		–	–	–	–	–	–	–	–	–	–	–	0.03	–
Natriuretic Peptides (NP)		–	–	–	–	–	–	–	–	–	–	–	–	–
Bradykinin (BK)		–	–	–	–	–	–	–	–	–	–	–	–	–
Waprin		–	–	–	–	–	–	–	–	–	–	–	–	–
Cobra Venom Factors (CVF)		–	–	–	–	–	–	–	–	–	–	–	–	–
Nucleases (DNAse, Rnase, Phosphodiesterase)		–	–	–	–	–	–	–	–	–	–	–	–	–
Carboxy peptidase		–	–	–	–	0.04	–	–	–	–	–	–	–	–
Others^d^		–	–	–	1.6	–	–	6.47	0.2	0.7	–	–	–	–

Venom Toxins	Country	EI-B	EI-N	EI	Bng	Bng	Chn	Twn	Twn	Tha	Tha^a^	Idn	Myn
	Year	2018	2018	2021	2019	2023	2018	2018	2018	2020	2022	2020	2009
	Reference No.	10	10	9	2	11	12	13	12	14	15	14	16
Phospholipase A2 (PLA2)		22.19	21.5	35.43	47.5	26	22.18	47.50	24.47	37.92	28.36	48.37	Yes
Snake Venom Metalloprotease (SVMP)		19.79	17.71	1.35	12.6	9	8.92	9.2	5.86	3.04	21.64	2.15	Yes
Snake Venom Serine protease (SVSP)		13.89	14.3	8.08	14.2	15	13.61	19.1	17.51	18.07	27.84	22.41	Yes
Phosphodiesterase (PDE)		0.74	0.5	–	0.9	–	0.25	0.9	0.31	0.16	–	0.89	–
5'-nucleotidase (5'NT)		0.96	0.5	–	0.8	–	0.82	–	–	1.85	–	0.18	–
L-Amino Acid Oxidase (LAAO)		1.67	1.5	1.28	2.5	3	5.95	–	–	–	2.59	1.47	Yes
Snaclec/C-Type Lectins/C-Type Lectin Like (CTL)^b^		11.1	12.05	4.8	6.8	17	16.89	5.2	16.52	10.64	3.79	1.03	Yes
Kunitz-type serine proteinase inhibitor-like protein (KSPI)		20.29	22.90	38.68	7.9	6	23.17	9.4	28.21	22.37	2.59	–	–
Cysteine-rich secretory protein (CRISP)		3.88	3.08	7.32	2.9	9	0.95	0.1	–	–	–	–	–
Vascular Endothelial Growth Factor (VEGF)		1.11	3.45	0.59	1.9	6	4.79	7.2	4.84	5.42	1.6	–	Yes
Nerve growth factor (NGF)		1.55	0.73	2.47	0.5	3	2.11	0.3	2.13	0.52	4.59	0.85	–
Snake Venom Metalloproteinase Inhibitor (SVMPi)		–	–	–		–	–	–		–	–	–	–
Aminopeptidase (AP)		0.46	–	–	0.04	–	0.35	–	0.15	–	–	–	–
Disintegrin/Disintegrin-like (Dis)^c^		2.2	1.38	–	1.3	3		1.2	–	–	–	6.21	–
Aminotransferase (AMT)		–	–	–	–	–	–	–	–	–	–	–	–
Hyaluronidase (HA)		0.1	0.05	–	–	–	–	–	–	–	0.43	–	–
Phospholipase B (PLB)		–	0.06	–	–	–	–	–	–	–	2.24	–	–
Glutaminyl cyclase (GC)		0.07	0.38	–	0.03	–	–	–	–	–	–	–	–
Neurotoxic Three-FingerTtoxin (N-3FTx)		–	–	–	–	–	–	–	–	–	–	–	–
Cytotoxic Three-Finger Toxin (C-3FTx)		–	–	–	–	–	–	–	–	–	–	–	–
Natriuretic Peptides (NP)		–	–	–	–	–	–	–	–	–	1.39	–	–
Bradykinin (BK)		–	–	–	–	–	–	–	–	–	0.59	–	–
Waprin		–	–	–	–	–	–	–	–	–	0.19	–	–
Cobra Venom Factors (CVF)		–	–	–	–	–	–	–	–	–	1.60	–	–
Nucleases (DNAse, Rnase, Phosphodiesterase)		–	–	–	–	–	–	–	–	–	0.6	–	–
Carboxy peptidase (CP)		–	–	–	–	–	–	–	–	–	–	–	–
Others ^d^		–	–	–	0.1	3	–	–	–	–	–	16.44	–

References: 1. Tan et al. 2015. 2. Pla et al. 2019. 3. Faisal et al. 2021. 4. Sharma et al. 2015. 5. Kalita et al. 2018c. 6. Mukherjee et al. 2016. 7. Faisal et al. 2018. 8. Kalita et al. 2017. 9. Senji Laxme et al. 2021. 10. Kalita et al. 2018b. 11. Yasmin et al. 2024 12. Tan et al. 2018. 13. Sanz et al. 2018. 14. Lingam et al. 2020. 15. Saethang et al. 2022 16. Risch et al. 2009

*SL* Sri Lanka, *SI* South India, *Pak* Pakistan, *WI* West India, *CI* Central India, *EI* East India, *EI-N* East India-Nadia, *EI-B* East India-Burdwan, *Bng* Bangladesh, *Chn* China, *Twn* Taiwan, *Tha* Thailand, *Idn* Indonesia, *Myn* Myanmar

^a^The values provided are adjusted to make the combined total amount of enzymatic and nonenzymatic group toxins equal to 100%

^b^Includes Snaclec, C-type lectins, and C-type lectin-like toxins combined

^c^Includes disintegrin and disintegrin-like toxins combined

^d^Includes unidentified proteins, hypothetical proteins, and all other proteins

**Fig. 1:**
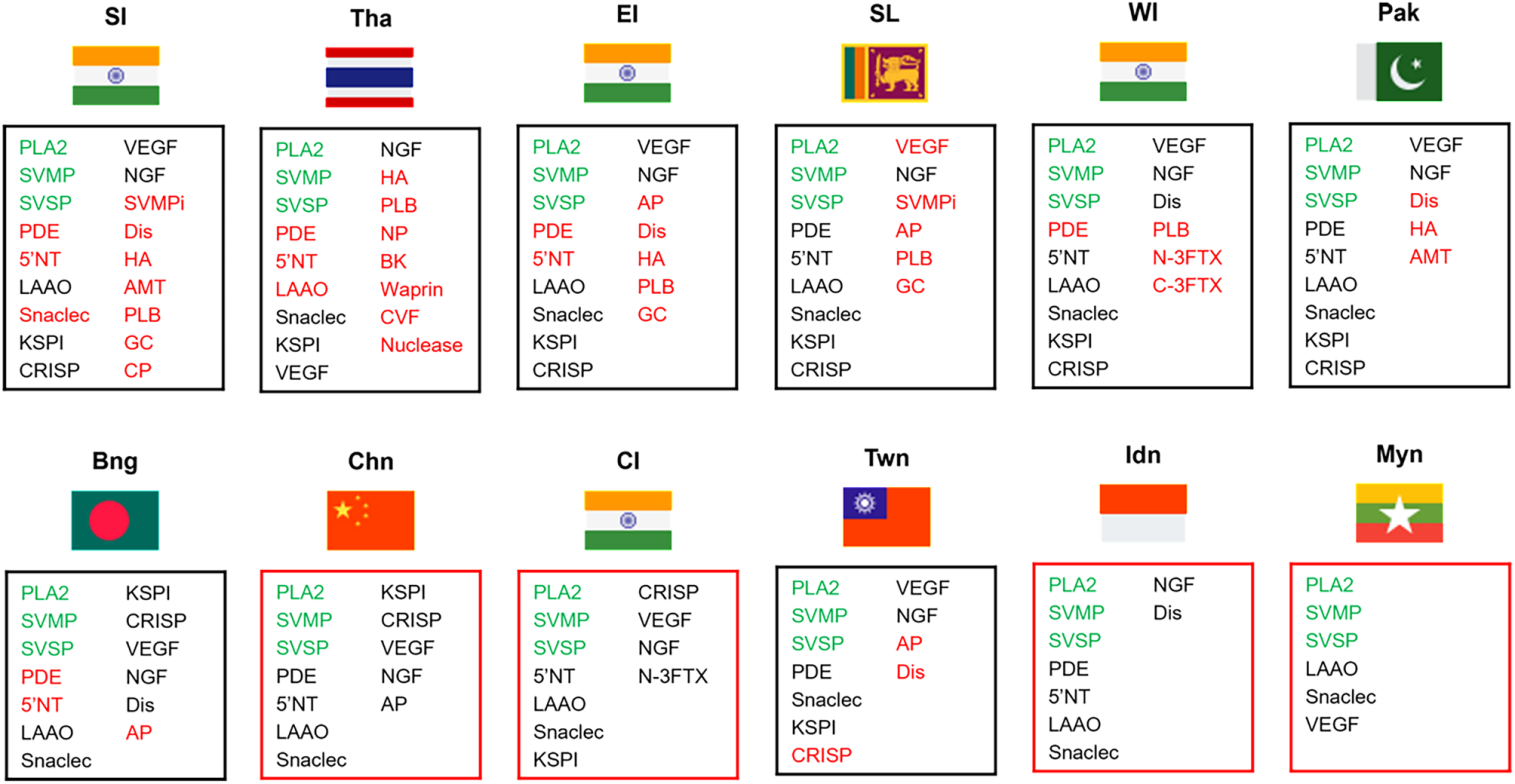
Venom proteomic profile of Russell’s viper (*Daboia russelii*) across Southeast Asia. The black boxes depict proteomic profiles from multiple studies, and the red boxes represent profiles from a single study. Texts in green indicate toxins common across Southeast Asia, black represents toxins common in that specific region, and red highlights toxins with discrepancies (refer to Table 1 for further details). The flag icons were obtained from Icons8 (https://icons8.com). *SI* South India, *Tha* Thailand, *EI* East India, *SL* Sri Lanka, *WI* West India, *Pak* Pakistan, *Bng* Bangladesh, *Chn* China, *CI* Central India, *Twn* Taiwan, *Idn* Indonesia, *Myn* Myanmar, *PLA2* Phospholipase A2, *SVMP* Snake Venom Metalloproteinase, *SVSP* Snake Venom Serine Protease, *PDE* Phosphodiesterase, *5ʹNT* 5*ʹ* Nucleotidase, *LAAO* L Amino Acid Oxidase, *Snaclec* Snake C-type Lectins, *KSPI* Kunitz Serine Protease Inhibitor, *CRISP* Cysteine Rich Secretary Protein, *VEGF* Vascular Endothelial Growth Factor, *NGF* Nerve Growth Factor, *SVMPi* Snake Venom Serine Metalloprotease inhibitor, *AP* Aminopeptidase, *HA* Hyalurinase, *Dis* Disinitegrins/Distinitegrin Like, *AMT* Aminotransferase, *PLB* Phospholipase B, *GC* Glutaminyl cyclase, *N-3FTx* Neurotoxic Three Finger Toxin, *C-3FTx* Cytotoxin Three Finger Toxin

Moreover, the presence of venom toxins within the same region was found to vary across studies. For instance, a couple of studies conducted in South Indian venom (Kalita et al. 2018c; Pla et al. 2019) reported the presence of PDE and 5ʹNT but was absent in the study performed by Faisal et al. (2021).This could be due to different strategies employed in the study or variation in species. A particular study in which venoms from two districts of the same state (West Bengal, India) also showed variation in toxin presence (Kalita et al. 2018b). This could be attributed to the diversity in the snake venom profile. Figure 1 shows the variation in the composition of venom across Southeast Asia. Taken together, these proteomic studies underscore the variation and diversity among them, and conducting proteome profile studies using standard procedures will help us better correlate the findings. In addition, these toxins clustered into different families are known to be present in numerous proteoforms.

## Phospholipase A2

The PLA2 enzyme hydrolyses glycerophospholipids at the sn-2 ester position of the glycerol backbone, thus releasing arachidonic acid and lysophospholipids. The PLA2 superfamily is categorized into 16 groups that are further classified into six subfamilies depending on their physiological location, substrate specificity, and heterogenicity: secreted PLA2s (sPLA2s), cytosolic PLA2s (cPLA2s), Ca^2+^-independent PLA2 (iPLA2), lysosomal PLA2s (LPLAs2), platelet-activating factor acetylhydrolase PLA2s (PAF-ADH PLA2s) and adipose tissue-specific PLA2s (AdPLA2s) (Schaloske and Dennis 2006; Dennis et al. 2011). All snake venom falls under the category of secreted PLA2 and is distributed in two groups, namely, group IA PLA2, which involves the Elapidae family, and group IIA PLA2, which comprises the Viperidea family of snakes. The PLA2 of Russell’s viper falls under the category of group IIA (Dennis et al. 2011).

**Fig. 2:**
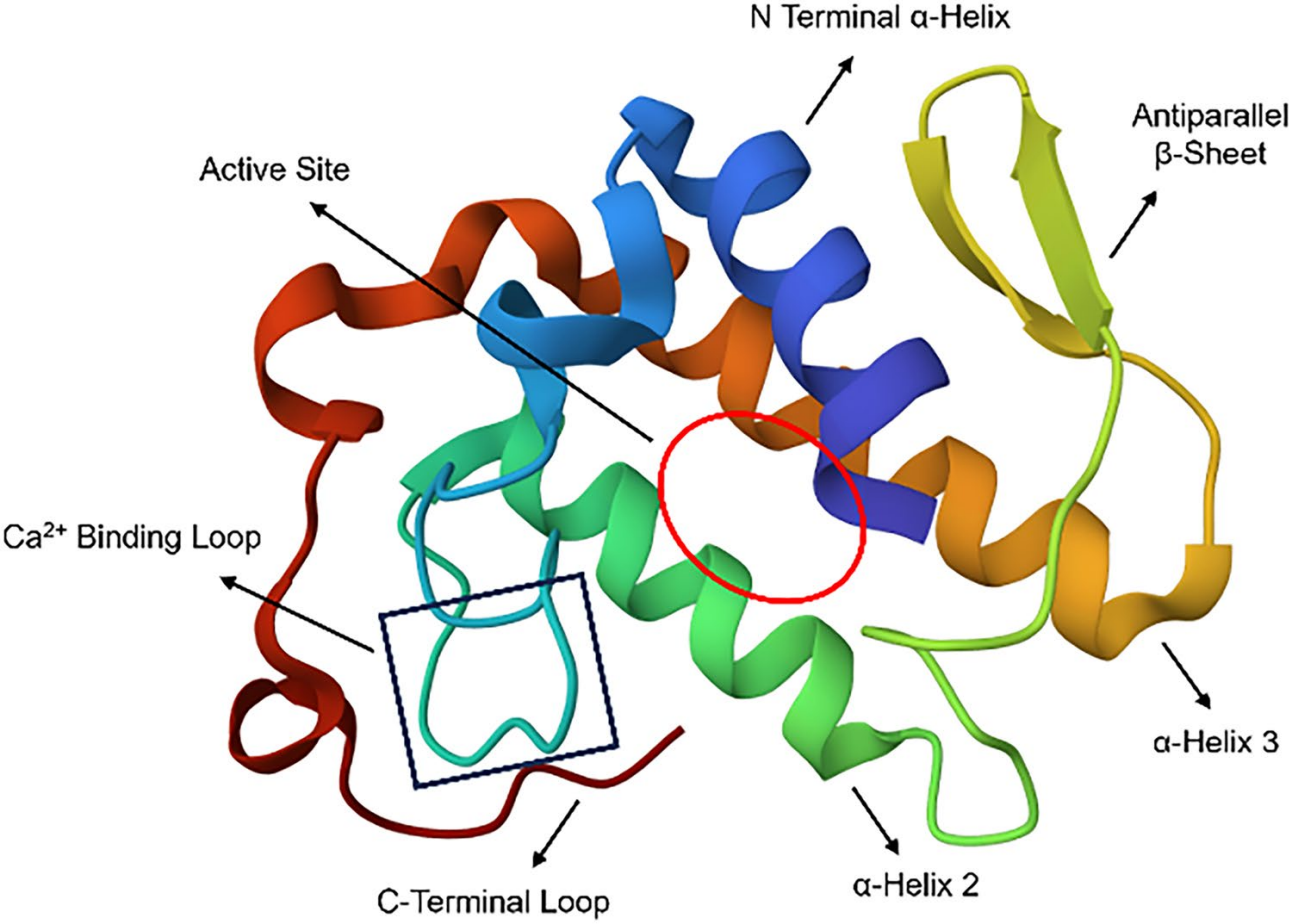
Structure of PLA2.PLA2 (PDB ID: 1FB2) contains an N-terminal α helix, two disulfide-connected α helices (α-helix 2 and α-helix 3), a double-stranded antiparallel beta-sheet, a Ca^2+^-binding loop, and a C-terminal loop. The active site is present between α-helix 2 and α-helix 3

The snake venom protein PLA2 is a small protein ranging from 120 to 135 amino acid residues and weighs approximately 14 to 18 kDa. They possess 6–7 disulfide bonds for their stabilization and require calcium for their catalytic activity. The snake venom PLA2 structure is characterized by an N-terminal alpha helix, two disulfide-connected alpha helices where the catalytic dyad is located, a double-stranded antiparallel beta-sheet, a Ca^2+^-binding loop, and a flexible C-terminal loop, as shown in Fig. 2 (Castro-Amorim et al. 2023). The hydrophilic residues are present in the region between the two antiparallel alpha helices, while hydrophobic residues occupy the core. However, the polar residues (His48, Asp49, Tyr52, Tyr73, and Asp99) that comprise the catalytic dyad and its hydrogen bond network are buried in the protein core. The hydrophobic channel composed mainly of Trp19, Ile9, Phe5, and Leu2 residues facilitates phospholipid binding and shields the His48/Asp99 dyad from the solvent environment. Furthermore, a Ca^2+^-binding loop is present that is characterized by Tyr28, Gly32, and Gly30. This loop in tandem with the β-carboxyl group of Asp49 aids in the binding of the cofactor (Castro-Amorim et al. 2023). Secreted PLA2s follow two catalytic mechanisms, namely, the single water mechanism (Verheij et al. 1980; Yu et al. 1986) and the assisted water mechanism (Yu et al. 1986). Furthermore, snake venom PLA2 is known to exhibit a phenomenon called interfacial activation, wherein the catalytic activity of the enzyme increases many-fold upon shifting itself from a dispersed form to an aggregate (Scott et al. 1990). However, the molecular mechanism behind this property of PLA2 is not understood.

## Mechanism of action

**Fig. 3:**
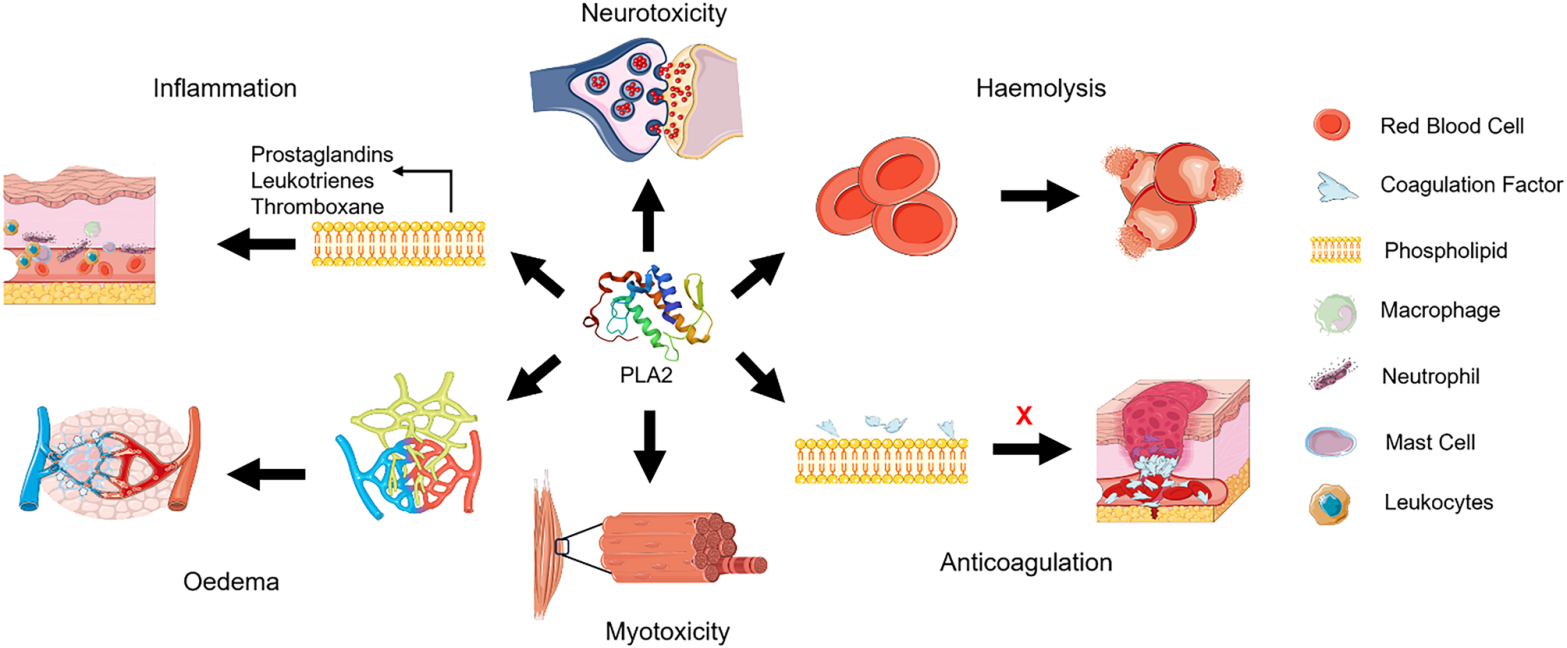
Mechanism of action of PLA2. *Hemolysis* PLA2 causes hemolysis either by red blood cell (RBC) (direct) or phospholipid (indirect) hydrolysis. *Anticoagulation* PLA2 interacts with phospholipids or coagulation factors and prevents coagulation. *Myotoxicity* PLA2 causes membrane disruption, Ca^2+^ influx, and mitochondrial damage, leading to irreversible muscle damage. *Oedema* PLA2 affects the lymphatic system, resulting in fluid accumulation. *Inflammation* PLA2 hydrolyses phospholipids, leading to the generation of lipid mediators and resulting in inflammation. *Neurotoxicity* PLA2 induces Ca^2+^ release, inhibits choline uptake, and suppresses acetylcholine desensitization-mediated synaptic activity. The image materials were obtained from Servier Medical Art and modified (https://creativecommons.org/licenses/by/4.0/)

PLA2 is known for causing a range of pharmacological effects in victims, including hemolysis, anticoagulation, oedema, myotoxicity, and neurotoxicity. Figure 3 depicts the various pharmacological effects of PLA2. PLA2 is reported to induce hemolysis by directly hydrolyzing the red blood cell (RBC) membrane or indirectly via lysophospholipids released upon hydrolysis of phospholipids (Urs et al. 2014). In addition to lysophospholipids, the phospholipid hydrolysis releases free fatty acids (arachidonic acid) that eventually stimulate the formation of lipid mediators such as prostaglandins, leukotrienes, and thromboxane. These lipid mediators induce inflammation via their enzymatic activity. PLA2 also affects the lymphatic system, resulting in fluid accumulation in the tissues, causing oedema (Mora et al. 2008). Furthermore, the anticoagulation effects exhibited by PLA2 either follows an enzymatic or nonenzymatic pathway. In the former, PLA2 hydrolyses the procoagulant plasma phospholipids required for the coagulation cascade, while in the latter, PLA2 binds to the coagulation factors thus making them unavailable for coagulation (Saikia et al. 2011).

Apart from these, PLA2 exhibits myotoxicity by binding to the sarcolemma and causing membrane disruption, resulting in loss of permeability. This disruption causes membrane depolarization and an influx of Ca^2+^. An increase in Ca^2+^ concentration leads to hypercontraction of myofilaments and activation of calpains, which degrade cytoskeletal components. Furthermore, mitochondrial Ca^2+^ uptake results in mitochondrial swelling, cristae disorganization, hydroxyapatite crystal formation, flocculent density, and permeability transition pore formation, eventually impairing membrane function. In addition, cytosolic PLA2 activation results in further membrane hydrolysis, culminating in irreversible damage (Gutiérrez and Ownby 2003; Montecucco et al. 2008).

Neurotoxicity is another pharmacological effect demonstrated by PLA2, wherein it increases the release of Ca^2+^ and presynaptic arachidonic acid from the endoplasmic reticulum. The latter activates protein kinase C (PKC), which amplifies the release of acetylcholine at the presynaptic terminal and activates the fusion protein complex. In addition, arachidonic acid inhibits the choline uptake transporter, reducing the supply of choline required for acetylcholine synthesis at the presynaptic terminal. Furthermore, arachidonic acid interacts with SNARE proteins, causing a biphasic effect resulting in transient facilitation and persistent depression. It also causes synaptic vesicle depletion and receptor inactivation, leading to the suppression of pre and postsynaptic activity. Sampat et al. (2023) reviewed the mechanism of the pharmacological effects of PLA2 in detail. In general, PLA2 exists in various isoforms and is reported to have different pharmacological effects (Schaechter and Benowitz 1993; Ranawaka et al. 2013; Šribar et al. 2014).

## PLA2 isoforms

**Fig. 4:**
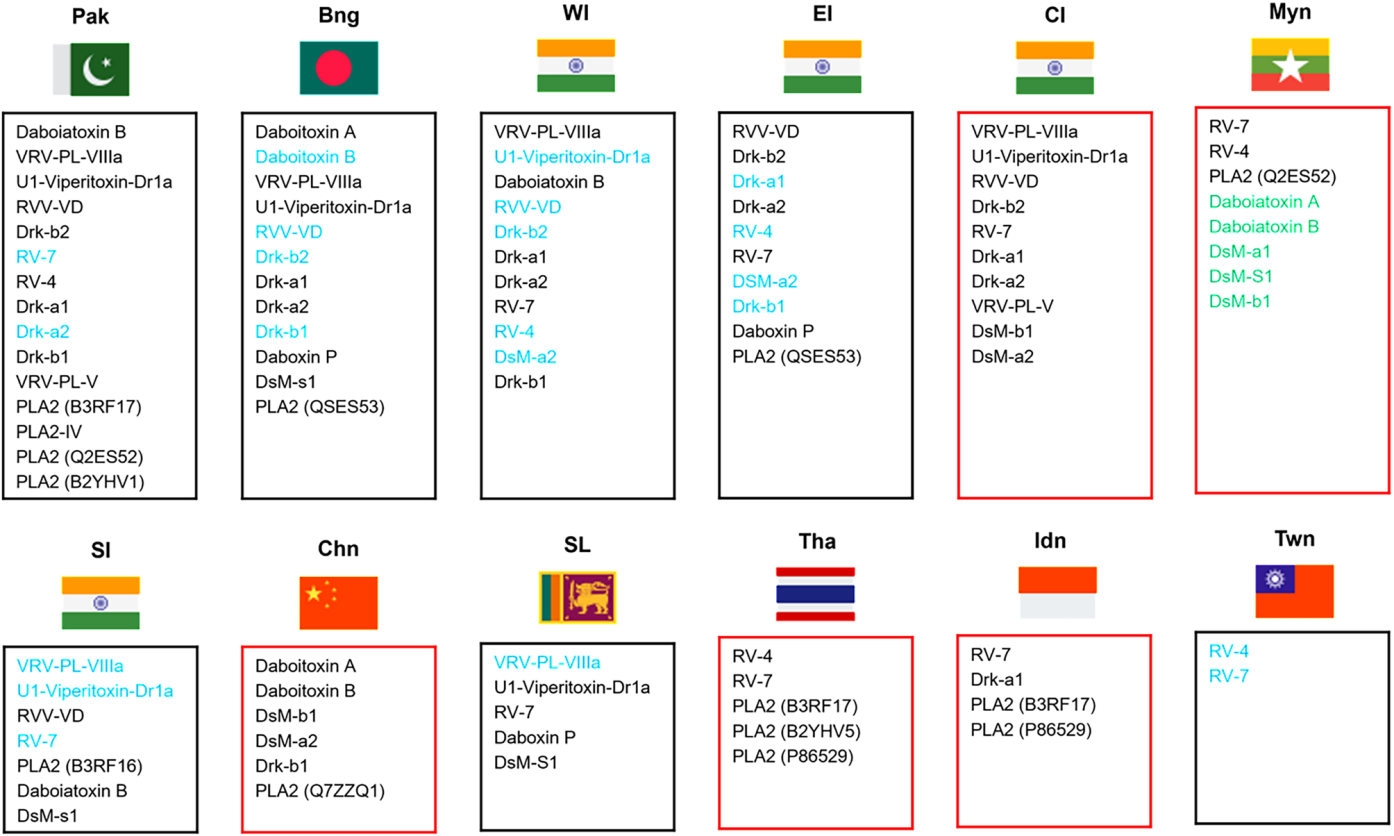
PLA2 isoforms of Russell’s viper (*Daboia russelii*) across Southeast Asia. The black boxes depict isoforms from multiple studies, and the red boxes represent isoforms from a single study. Texts in blue represent isoforms common in that specific region, black highlights isoforms with discrepancies, and green indicates isoforms not reported in the proteomic profile but in other studies (refer to supplementary Table S1 for further details). The flag icons were obtained from Icons8 (https://icons8.com). *Pak* Pakistan, *Bng* Bangladesh, *WI* West India, *EI* East India, *CI* Central India, *Myn* Myanmar, *SI* South India, *Chn* China, *SL* Sri Lanka, *Tha* Thailand, *Idn* Indonesia, *Twn* Taiwan

Proteomic studies of the venom of Russell’s viper across different biogeographical regions have revealed that PLA2 is present in numerous isoforms (Fig. 4 and Table S1). Tsai et al. (1996) proposed the existence of two Russell’s viper types, distinguished based on the presence of either serine (S) or asparagine (N) residues at the N-terminus of PLA2 isoenzymes. Various research groups have separated these isoforms and studied their enzymatic and pharmacological properties. PLA2 VRV-PL-VIIIa is a basic isoform present in the venom of the Indian subcontinent, with high abundance in the venom of the South Indian and Sri Lankan venoms (Pla et al. 2019; Faisal et al. 2021). It exhibits neurotoxicity, myotoxicity, oedema, anticoagulant activity, and hemorrhage. A study highlighted the presence of lysine residues at the active site to be vital for lung hemorrhage activity (Uma and Gowda 2000). Similarly, U1-viperitoxin-Dr1a is another abundant PLA2 toxin found in the southern part of India and Sri Lanka. It exhibits 89% identity with another isoform, PLA2 VRV-PL-V that differs at amino acid residues 58, 61, 97, and 98 from the N-terminus. U1-viperitoxin-Dr1a is reported to showcase a neurotoxic effect at the presynaptic terminal with mild potency (Silva et al. 2017). Along with PLA2 VRV-PL-VIIIa (also known as U1-viperitoxin-Dr1b), U1-viperitoxin-Dr1a aids in the mild myotoxic effect of the venom. While various research groups have studied the properties of PLA2-VRV-PL-V by isolating it from Russell’s viper venom from South India (Jayanthi et al. 1989; Dharmappa et al. 2010; Sudarshan and Dhananjaya 2014), its presence has not been reported in proteomic studies conducted from the venom of South India (Faisal et al. 2021; Kalita et al. 2018c; Pla et al. 2019) or Sri Lanka (Faisal et al. 2021; Pla et al. 2019; Tan et al. 2015). However, its abundance was observed in the venom from Pakistan (Pla et al. 2019). It demonstrates oedema, anticoagulation effects, and presynaptic neurotoxicity (Jayanthi et al. 1989).

Basic Drk-b2 is the major PLA2 isoform found in Bangladesh, accounting for 27.1% of the total venom proteome (Pla et al. 2019). Although this proteoform has been identified in venoms from Pakistan and India, its composition varies (Faisal et al. 2018; Kalita et al. 2018b; Senji Laxme et al. 2021). Despite its prevalence, the pharmacological effects of Drk-b2 have not been extensively studied, and investigating these effects could provide valuable insights into this isoform. RVV-VD has 84% identity and is one of the closest homologs of basic Drk-b2. It is distributed across Pakistan (Mukherjee et al. 2016; Faisal et al. 2018), Bangladesh (Pla et al. 2019; Yasmin et al. 2024), and India (Faisal et al. 2018; Kalita et al. 2018b; Senji Laxme et al. 2021). Despite having poor catalytic activity and lethality, this basic PLA2 isoform exhibits significant anticoagulant activity. It has been postulated that the anticoagulant site of PLA2-RVV-VD lies between residues 53 and 76 and is rich in lysine residues, and its potency is mainly attributed to the presence of Lys 56 and Lys 67 (Carredano et al. 1998).

Studies have highlighted the importance of conserved residues in demonstrating specific properties and underscoring the diversity among isoforms. Although DsM-bI and Drk-bI share 97% identity, DsM-bI exhibited neurotoxic and lethal properties compared to Drk-bI. This difference in physiological properties could be attributed to Thr41, which is conserved in all neurotoxic and myotoxic PLA2 isoforms, while PLA2 isoforms exhibit lower levels of the toxicity feature Ser41 (Tsai et al. 2007). The PLA2 isoform DsM-b1 isolated from *Daboia siamensis* of Myanmar was reported in the proteomic profile of venom from China and India (Madhya Pradesh) (Senji Laxme et al. 2021). Leu 2 is highly conserved and reported to be vital for substitute binding. Compared to neurotoxic and lethal Drk-a1, DsM-aI, with its 2nd residue substituted with Phe, demonstrated a 40–45-fold reduction in lethality and catalytic rate (Tsai et al. 2007). Furthermore, both Drk-aI and Drk-a2 isoforms are observed across venoms from Pakistan, Bangladesh, and different parts of India, while only Drk-a1 is present in Indonesia (Mukherjee et al. 2016; Faisal et al. 2018; Pla et al. 2019; Lingam et al. 2020; Senji Laxme et al. 2021).

Daboiatoxin, a lethal PLA2 toxin from the venom of *Daboia russelii siamensis* (Myanmar), shares homology with viperotoxin from *Daboia russelii formosensis* (Taiwan) and vipoxin from Bulgarian sand *vipera ammodytes meridionalis* (Maung-Maung-Thwin et al. 1995)*.* Daboiatoxin is a heterodimer of two PLA2 chains (A and B) that has potent neurotoxic, myotoxic, and cytotoxic effects. It also demonstrated oedema-inducing and indirect haemolytic activity but lacked haemorrhagic activity. Chain A is less lethal and has lower PLA2 activity than Chain B. The former acts as an inhibitor, thus reducing the enzymatic activity of Chain B by 30% (Gopalan et al. 2007). While only Daboiatoxin chain B was identified to be present in Russell’s viper venom from Pakistan (Mukherjee et al. 2016; Faisal et al. 2018) and western India (Kalita et al. 2017), studies conducted on the venom from Bangladesh (Yasmin et al. 2024) and China reported the presence of both chains. However, a study by Pla et al. (2019) reported the presence of Daboiatoxin B alone in the venom of Bangladesh. Despite Daboiatoxin and DsM-aI having identical N-terminal sequences, the latter exhibited less toxicity, and the reason behind this inconsistency is yet to be explored (Tsai et al. 2007). Furthermore, the Daboiatoxin purified from Myanmar had less homology with the Thai Russell’s viper.

On the other hand, Daboxin P, isolated from *Daboia russelii russelii,* is nontoxic but shows strong PLA2 and anticoagulant properties. It demonstrated anticoagulant activity by targeting both factors X and Xa, which are involved in the coagulation cascade (Sharma et al. 2016). Moreover, it inhibits platelet aggregation mediated by the agonist thrombin (Yasmin et al. 2023). Although Daboxin P is commonly present in venom from Eastern India (Kalita et al. 2018b) and Bangladesh (Yasmin et al. 2024), the proteomic profile of Sri Lankan venom (Pla et al. 2019) has also revealed its presence.

Protein characterization and DNA cloning studies performed on Taiwan Russell's viper (*Vipera russelli formosensis*) revealed that the PLA2 isoforms RV-4 and RV-7 share 72% sequence identity with Bulgarian *Vipera a. ammodytes’* vipoxin and vipoxin inhibitors (Warrell 1989). RV-7 is nontoxic and shows low enzymatic activity because the presence of acidic residues at positions 7, 17, 59, 114, and 119 weakens its binding to the substrate. RV-7 and RV-4 exist as dimers in crude venom, with RV-7 potentiating the neurotoxicity and lethality of RV-4 while inhibiting its enzymatic activity. RV-4 and RV-7 are similar to Thai Russell’s vipers PLA2S1 and PLA2S2, respectively. Although both of these species have been categorized into different subspecies, their gene sequence analysis suggested a close evolutionary relationship between them. In addition, the PLA2-Drka1 isoform isolated from *Daboia siamensis* in Myanmar, which is highly neurotoxic and lethal, shares 91% identity with RV-7. Substituting approximately 10 amino acid residues in RV-7 could change its pharmacological properties (Tsai et al. 2007). Other studies conducted on Taiwan Russell’s viper proteomic profiles (Sanz et al. 2018; Tan et al. 2018) also reported the presence of RV7 and RV4, exhibiting consistent results with Taiwan PLA2 reported earlier. Furthermore, the abundance of RV7 was high in Thai Russell’s viper compared to all other proteomic profiles (Lingam et al. 2020). Proteomic profiles showed the presence of DsM-S1 in the venom of southern India (Faisal et al. 2021), Sri Lanka (Faisal et al. 2021), and Bangladesh (Yasmin et al. 2024). In addition, other PLA2 isoforms were present in trace amounts. Thus, the proteomic profile of PLA2 from Russell’s viper venom from different biogeographical regions has shown diversity in its composition and demonstrated differences in its pharmacological properties.

## Antivenoms

The administration of antivenom is the primary treatment for snakebite envenomation. They are mainly polyclonal and are categorized into three types: whole IgG, F(ab’)2, and Fab, which weigh approximately 150 kDa, 100 kDa, and < 50 kDa, respectively. These antibodies possess different pharmacodynamics and pharmacokinetics and have been extensively reviewed (Nikapitiya and Maduwage 2018). However, there are numerous challenges associated with commercial antivenoms. One of them is their ineffectiveness across geographical boundaries due to inter- and intraspecies variation in venom. The antivenoms manufactured in India are administered to victims in neighboring countries such as Sri Lanka and Pakistan, and reports have claimed their limited effectiveness. For instance, Indian polyvalent antivenoms from VINS Bioproducts and Bharath Serum and Vaccines, only partially inhibited the neurotoxic effect and failed to prevent in vitro myotoxicity of *Daboia russelii* from Sri Lanka. Similarly, the antivenom from the VINS bioproduct had poor potency against *Daboia russelii* from Pakistan. As a result, various region-specific antivenoms have been developed and investigated. Studies lead by Choo Hock Tan and team revealed that Pakistani viper antivenom (PVAV) possesses better immunoreactivity, efficacy, and binding potency against *Daboia russelii* from Pakistan (Lim et al. 2023, 2024). In addition, studies conducted by two independent groups on Sri Lanka-specific polyvalent antivenoms demonstrated better neutralization of the toxic and lethal effects exhibited by Russell’s viper than by Indian antivenom (Villalta et al. 2016; Patra et al. 2021). These results demonstrated that region-specific antivenoms had better efficacy.

Administering antivenom as early as possible is another major challenge. Administration of Indian antivenom to patients in Sri Lanka who presented with Russell’s viper bites failed to prevent or reverse neurotoxicity even though the venom concentration decreased. Another study demonstrated that preincubating Sri Lankan Russell’s viper venom with Indian polyvalent antivenom from Bharath Serums and Vaccines prevented myotoxicity but failed to reverse myotoxicity with delayed administration (Thakshila et al. 2022). Similarly, delayed administration of Chinese antivenom failed to neutralize the pharmacological effects of the venom (Lay et al. 2023). Furthermore, the addition of Chinese *Daboia siamensis* monovalent antivenom (DsMAV) or Thai-DsMAV with *Daboia siamensis* venom from China beforehand prevented presynaptic neurotoxicity and myotoxicity in vitro (Lay et al. 2022). Likewise, another study demonstrated that regional monovalent and polyvalent antivenoms failed to prevent the capillary leak syndrome induced by Russell’s viper upon delayed administration (Lingam et al. 2021). The distribution of venom toxins to specific tissues is the major reason behind ineffective neutralization by antivenoms, resulting in irreversible tissue damage.

Besides commercial polyvalent antivenoms, monovalent antivenoms raised against specific *Daboia russelii* species have also been explored. DsMAV-Thailand, raised against Thailand *Daboia russelii*, showed strong immunoreactivity towards Russell’s viper venom native to Thailand (Lingam et al. 2020). China’s Ds-MAV prevented the myotoxicity and neurotoxicity of Chinese Russell’s viper venom (Lay et al. 2022). Likewise, DsMAV-Taiwan demonstrated immunoreactivity and effectively neutralized the venom lethality of Taiwan Russell’s viper. Taken together, these studies emphasize the capability of species-specific monovalent antivenoms to neutralize the pharmacological effects compared to polyvalent antivenoms. These monovalent antivenoms raised against snakes native to a particular region were also effective against the venom of other regions that share conserved antigenicity of vital toxins. For instance, the monovalent antivenom (VINS Bioproducts Limited, India) produced against crude Russell’s viper venom showed better cross-reactivity and neutralization of procoagulant/anti-coagulant activity against Pakistani Russell’s Viper (Captive) venom than did the polyvalent antivenom from Bharat Serum and Vaccines Limited, India (Mukherjee et al. 2016). Similarly, DsMAV-Thailand was effective against venom from China (Lay et al. 2022) and Indonesia (Lingam et al. 2019, 2020), while DsMAV-Taiwan has shown to be effective against Chinese Russell’s viper. These studies underscore the efficacy of monovalent antivenoms over polyvalent antivenoms in neutralizing venom toxicity.

The next major problem with antivenom administration is the inability of antivenoms to neutralize lower-molecular-weight venom toxins. Studies conducted using commercial Indian polyvalent antivenoms have demonstrated the potential to neutralize high-molecular-mass toxins but have shown poor immune cross-reactivity and neutralization of low-molecular-weight toxins (< 20 kDa) against venoms from southern (Kalita et al. 2018c), eastern (Kalita et al. 2018b), and western (Kalita et al. 2017) India. In another study, Indian VPAV showed low binding efficacy against Pakistani Russell’s viper venom from the wild, with low molecular weight fractions exhibiting weak antivenom binding activity (Faisal et al. 2018). Similar effects were also observed in Sri Lankan Russell’s viper (Faisal et al. 2021). Studies have reported that even monovalent antivenoms poorly neutralized the low-molecular-mass proteins. Lingam et al. (2020) showed DsMAV-Thailand to possess strong immunorecognition towards the major protein fractions but demonstrated low immunoreactivity towards low-molecular-weight proteins in the venom of Indonesia and Thailand. Likewise, monovalent antivenom from VINS displayed poor cross-reactivity and failed to neutralize the pharmacological effects demonstrated by lower molecular mass toxins (Mukherjee et al. 2016).

Collectively, these studies underscore the drawbacks of both monovalent and polyvalent antivenoms, emphasizing the importance of neutralizing specific low-molecular-mass toxins. They highlight the immediate requirement for next-generation antivenoms wherein low-molecular-weight toxin-specific inhibitors are designed. Snake venom consists of several low-molecular-weight toxins below 20 kDa such as PLA2, VEGF, NGF, KSPI, Snaclec, and disintegrins. Among these, PLA2 is an abundant low molecular mass toxin, and designing specific inhibitors against this toxin is crucial.

## PLA2 inhibitors

To develop next-generation antivenoms, various molecules such as plant derivatives, aptamers, peptides, nanoparticles, and small molecules have been investigated for their ability to neutralize individual venom toxins. Studies have investigated the use of plant extracts and plant-based derivatives to overcome the toxicity induced by PLA2. For example, extracts of *Tamarindus indica Linn* (Maung and Lynn 2012)*, Andrographis serpyllifolia* (Hansiya and Geetha 2021), and *Cyanthillium cinereum* (Suji et al. 2023) were able to neutralize the PLA2 activity and hemotoxicity effects of Russell’s viper. The aqueous extract of *Mangifera indica* reduced the PLA2 activity and oedema induced by VRV-PL-VIIIa (Dhananjaya et al. 2011). Rutin, a flavonoid found in plants, was reported to inhibit PLA2 from *Daboia russelii* and *Crotalus atrox* (Lindahl and Tagesson 1997)*.* AIPLAI, a compound isolated from *Azadrirachta indica* (Neem) leaves suppressed the enzymatic and pharmacological effects of *Daboia russelii* PLA2 (Mukherjee et al. 2008). In addition to demonstrating dose-dependent anti-hemolytic activity against the PLA2 isoforms VRV-PL-V and VRV-PL-VIIIa, diosmin, derived from *Oxalis corniculata L.*, also mitigated their myotoxicity and cardiotoxicity effects (Kiran et al. 2024). Plant derivatives such as butein, mimosine, and bakuchiol neutralize the PLA2 activity exhibited by dapoxin P, and mimosine and bakuchiol additionally neutralize its anticoagulation activity (Devi et al. 2020).

Phenolic compounds have been shown to exhibit good antivenomous properties. For instance, 2-hydroxy-4-methoxy benzoic acid isolated from the root extract of *Hemidesmus indicus* exhibited antivenomous properties against *Daboia russelii* (Alam et al. 1994). Another group (Nargotra et al. 2011) screened various natural and synthetic PLA2 inhibitors of *Daboia russelii* PLA2 via in silico docking and reported phenolic and substituted benzaldehyde compounds as major inhibitors. Notably, phenolic compounds possessing hydroxyl and methoxy groups in their benzene ring exhibited greater inhibitory potency by forming hydrogen bonds with the Asp49 and Gly30 residues of *Daboia russelii* PLA2 (Alam et al. 2016). Furthermore, pyrazolo[3,4- d] pyrimidine molecules were found to dock with enzymatic and anticoagulant regions of *Daboia russelii* PLA2 (Yadava et al. 2013). Another study reported that an imidazopyridine derivative formed π–π stacking interactions with Trp31 and amide–π stacking interactions with Gly32 (Anilkumar et al. 2015).

In addition, structural studies have provided insights into the interactions between PLA2 and various other compounds. An indole derivative (2-carbamoyl methyl-5-propyl-octahydro-indol-7-yl)-acetic acid) was reported to interact with the active sites and form hydrophobic interactions with the substrate binding channel of PLA2 from *Daboia russelii pulchella* (Balasubramanya et al. 2005). Similarly, alpha-tocopherol (vitamin E) interacts with active residues and forms a stable complex via interactions with hydrophobic channels (Chandra et al. 2002a). The alkaloids berberine from *Cardiospermum halicacabum* and aristolochic acid from *Aristolochia* species were also reported to interact with Russell’s viper PLA2 (Chandra et al. 2011, 2002d). Similarly, p-Coumaric acid, spermidine, corticosterone, resveratrol, and a gramine derivative were able to bind to the substrate binding region of PLA2 in *Daboia russelii pulchella species* (Shukla et al. 2015)*.* Compounds such as anisic acid and atropine possessing anti-inflammatory properties were found to bind to the substrate binding cleft of PLA2 of *Daboia russelii pulchella.* Similarly, various nonsteroidal anti-inflammatory drugs (NSAIDs), such as diclofenac and oxyphenbutazone (Singh et al. 2004), interacted with the substrate binding site, while indomethacin (Nagendra et al. 2009) interacted with regions vital for catalytic and anticoagulant activity. Besides, through structural analysis, peptides were also reported to bind the substrate binding sites of PLA2. For instance, a study conducted in PLA2 of *Daboia russelii pulchella* revealed the pentapeptide LAIYS to occupy the substrate binding cleft with the hydroxyl group of tyrosine interacting with the active site dyad of PLA2 and the hydrophobic residues of the peptide interacting with the hydrophobic channel of PLA2 (Chandra et al. 2002c). Another pentapeptide, FLSYK, was shown to interact with specific residues, such as Asp 49 and His 48 (Chandra et al. 2002b).

Furthermore, nanoparticles have emerged as effective inhibitors against *Daboia russelii* toxicity. Conjugating gold nanoparticles with andrographolide, a diterpenoid compound found in *Andrographis paniculate* reduced the damage induced by *Daboia russelii russelii* such as oedema, defibrination, hemorrhage, inflammation, and organ damage (Ghosh et al. 2019). Likewise, gold nanoparticle-conjugated 2-hydroxy-4-methoxybenzoic acid was revealed to possess potent neutralization ability (Saha and Gomes 2017). Furthermore, a study by Hingane et al. (2018) demonstrated silver nanoparticles to exhibit inhibitory effects on *Daboia russelii.* TiO2 nanoparticles also effectively neutralized the PLA2 activity and inflammation induced by *Daboia russelii* venom (Chakrabartty et al. 2019). These studies underscore the potential of nanoparticles to counter venom-induced toxicity.

Using aptamers is another approach that has been studied to neutralize venom toxins. These are single-stranded DNA or RNA oligonucleotides that can bind to their targets with high specificity and affinity. Devi and colleagues designed an aptamer against *Daboia russelii* toxin daboxin P. The results exhibited successful inhibition of the enzymatic and anticoagulant activity (Devi and Doley 2021). This emphasizes the ability of aptamers in neutralizing the toxic effects demonstrated by the venom toxins. However, further studies are required to improve their efficiency, thus paving the way for novel antivenom strategies.

Snakes have developed the ability to protect themselves from their own toxins via the presence of endogenous PLA2 inhibitory proteins (PLIs) in their blood serum. Depending on the structural features, motifs, and physiological properties, they are categorized into alpha, beta, and gamma PLIs (Campos et al. 2016). Various research groups have isolated these toxins and have reported their inhibitory potential against individual PLA2 toxins. For example, an endogenous PLA2 inhibitor termed as PLA2 inhibitor from Python (PIP) was purified from nonvenomous *Python reticulatus* and demonstrated to inhibit the effect of daboiatoxin (Thwin et al. 2000). Likewise, a PLI from cobra showed 10–100-fold greater inhibitory activity against PLA2-VRV-V (Inoue et al. 1997). These studies highlight the importance of snakes' own defensive proteins and how they can be employed to develop specific toxin inhibitors.

In recent times, small molecules have gained great attention due to their ability in neutralizing PLA2. Varespladib, a repurposed drug, and its orally available prodrug (varespladib methyl) have been reported to neutralize the effects of PLA2 demonstrated by various snake species, including *Daboia russelii* (Lewin et al. 2016). Varespladib was found to prevent the neurotoxicity and myotoxicity of Chinese Russell’s viper venom (Lay et al. 2023). A recent study showed that the co-administration of varespladib with Thai *Daboia siamensis* monovalent antivenom increased the efficacy of reversing presynaptic neurotoxicity (Lay and Hodgson 2024). Currently, the oral drug methyl varespladib has entered phase II clinical trials (National Library of Medicine NCT04996264; Carter et al. 2023). Similarly, another small molecule, 1,3,4-oxadiazole, inhibited the PLA2 VRV-PL-VIIIa isoform (Kameshwar et al. 2017). These studies highlight the importance of targeting low-molecular-weight toxins with specific inhibitors and administering them along with antivenoms to help improve their efficacy.

## Conclusion

Snakebite envenomation is a serious issue that needs to be addressed in tropical regions. With the WHO declaring snake venom envenomation as a neglected tropical disease, various attempts have been made to reduce morbidity and mortality. Russell’s viper is a medically important snake that has been extensively studied by various research groups. This study investigated the proteomic profiles of Russell’s viper venom across Southeast Asia. There is variation in the proteomic profile across geographical regions, but variation in the venom profile within a specific region could be due to the use of different approaches. Studying the proteome profile of Russell’s viper venom across Southeast Asia using standard methodology would help us obtain a detailed picture. Furthermore, having genomic and transcriptomic data for all of Russell’s vipers would aid in performing an in-depth analysis. Furthermore, the abundant toxin PLA2 and its isoforms have been well studied for their enzymatic and pharmacological roles for decades. These studies have highlighted the importance of conserved residues and the diversity among them. Currently, with antivenom administration being the only primary option for envenomation, the diversity of snake species and the presence of low-molecular-weight toxins have made antivenoms less effective. This has resulted in the need for the production of region-specific antivenoms wherein low-molecular-weight toxins are targeted using specific inhibitors. In future, the development of region-specific antivenoms fortified with low-molecular-weight toxin inhibitors could lead to effective treatments for snakebites.

## Supplementary Information

Below is the link to the electronic supplementary material.

Supplementary file1 (XLSX 22 KB)

## Data Availability

Data sharing is not applicable to this article, as no datasets were generated during the current study.
